# Giant cell tumor of the spine: A review of 9 surgical interventions in 6 cases

**DOI:** 10.4103/0019-5413.32047

**Published:** 2007

**Authors:** Shekhar Y Bhojraj, Abhay Nene, Sheetal Mohite, Raghuprasad Varma

**Affiliations:** Spine Clinic, P. D. Hinduja National Hospital and Breach Candy Hospital, Mumbai, India; * Spine Clinic, Cornelian, Mumbai, India; ** Spine Clinic, L. H. Hiranandani Hospital, Mumbai, India

**Keywords:** Giant Cell Tumor of the spine, GCT, radiotherapy

## Abstract

**Background:**

Giant cell tumor (GCT) of the spine is uncommon but most aggressive benign tumor of the spine with unpredictable outcome. We present our observation on six cases of GCT of the spine. We treated six patients with giant cell tumors (GCT) of the spine between 1993 and 2006. A total of nine surgical interventions were carried out. Four interventions were carried out in patients presented as ‘new’ cases, while five on recurrences from past GCT resections. All presented with cord compression and neurological deficits of varying grades. All patients also presented with clinical as well as radiological instability. Preoperative tissue diagnosis was available only in the five recurrences (tissue from the old resection). Posterior only (n=2), anterior only (n=4) and single-stage back and front (n=3) surgeries were carried out depending on the nature of the tumor.

**Results:**

Overall results were satisfactory, as all patients were symptom-free postoperatively. Two out of our four new patients had tumor recurrence and both needed repeat resection. Both have been disease-free at last follow-up.

**Conclusion:**

Surgical intervention is mandatory. Close follow up is needed for early diagnosis of recurrences.

Giant Cell Tumor (GCT) of the spine remains an intriguing and unpredictable entity. It is the most aggressive of the benign primary tumors of the spine, with a high predilection for recurrences. Most of the available literature reports small series, clearly indicating that it is not a common occurrence.^1^ ^2^

Spinal GCTs however, often present with the unique problem of spinal cord compression due to extension into the spinal canal. Also, as it is often situated on either side of the neural tissues, complete resection becomes surgically challenging and most often, marginal or intralesional excision with backup therapy has to be resorted to.

As with other sites, various treatment options have been described, ranging from surgical excision to adjuvant modalities like cement injection, phenol ablation, cryotherapy and radiotherapy. We report our experience with nine surgical interventions for six patients, over 13 years.

## MATERIALS AND METHODS

We retrospectively analyzed cases of spinal GCT that had been operated between 1993 and 2006. From the approximately 200 surgeries done for spinal tumors during this period, nine were for GCTs and formed our study group.

All data was extracted from hospital records, including preoperative and sequential postoperative clinical findings, radiological details and pictures and details of the status at last follow-up. Neurology was assessed by Frankel grading. X-rays and CT scans were studied for the presence or recurrence of tumor, instability and the status of the fusion and spinal implants. Quality of life questionnaires were not used. There were three males and three females in our group of six patients. Age ranged from 22 to 39 years. Mean follow-up was seven years (range - four to nine years).

Four patients presented as fresh cases. Two of these had a recurrence after their first surgery with us. One of the two had a third recurrence. Two patients presented with a recurrence of a spinal GCT operated elsewhere primarily. One of these had had two surgeries before presenting to us. All patients, presented with cord compression and neurological deficit. Three cases graded Frankel C at presentation and all others were Frankel D. All had clinical as well as radiological evidence of spinal instability.

Four of our six patients had GCTs in the thoracic spine [Table 1], one in the cervical spine (C2 level) and one in the lumbar spine (L3 level). One 30-year-old lady, who was being treated as spinal TB and had already undergone two surgeries at another institute, presented with residual tumor, with persistent instability and cord compression. One recurrent tumor presented with superadded infection. One of our cases had two recurrences over six years and thus needed three surgeries. He has been closely followed up clinically as well as with yearly CT scans and has had no recurrence since his third surgery eight years ago. Three of the recurrent tumors had implants from the previous surgery. One of these had fractured anterior implants and kyphosis. The other two had intact implants with tumor recurrence presenting as cord compression and neurological deficit.

**Table 1:** Summary of data of the six patients

Pt. No.	Age/sex	Level	1^st^ presentation	Recurrences	Surgery	Year of presentation	Neurological deficit at presentation
1	23/F	T11	Fresh case	One	1^st^: Posterior + anterior 2^nd^: anterior	1997	Frankel D at 1^st^ presentation, Frankel C at recurrence
2	27/F	T5	Fresh case	None	Anterior	1997	Frankel D
3	18/M	L3	Recurrent GCT with infection	One	Both posterior	1997	1. N/A (operated elsewhere)
							2. Frankel D
4	35/M	T3	Fresh case	None	Posterior	1998	Frankel D
5	36/M	C5	Fresh case	Two	1^st^ anterior	1993	1. Frankel C
					2^nd^ posterior + anterior	1994	2. Frankel D
					3^rd^ anterior	1999	3. Frankel D
6	26/F	T8	Recurrent GCT	One	Both posterior + anterior	2001	1. N/A (operated elsewhere)
							2. Frankel C

N/A = Not available, GCT - Giant cell tumor

On X-ray, all cases had lytic, expansile lesions, with a ‘soap bubble’ appearance. All the four ‘new’ cases in our series had an magnetic resonance imaging (MRI) as well as a computerized tomography (CT) scan preoperatively. In situations where MRI non compatible metallic implants were present from the primary surgery, CT scans alone were done preoperatively. The MRI gives an excellent delineation of the soft tissue component of the tumor mass, the exact location of the neural compression and to an extent, an idea of the tumor vascularity. A CT scan, on the other hand, reconstructs the bony anatomy very well to give a preoperative idea of the nature of reconstruction needed. Chest X-rays were done in all patients, to rule out an unusual but specific probability of lung metastasis.

Of the nine surgeries, the five that were done for recurrent tumors, had a histopathology report from the old surgery. Of the other four, two had had a preoperative fine needle aspiration cytology, which was inconclusive. In all these four cases without a preoperative diagnosis, an intraoperative frozen section was relied upon after making a preoperative diagnosis on radiology. In all the four cases, the radiological diagnosis of GCT matched with the frozen section and the eventual final histopathology.

**Figure 1:**
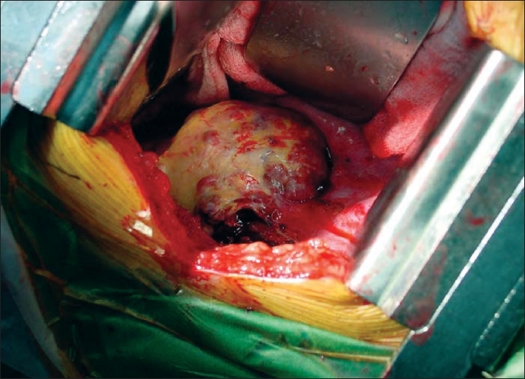
Intraoperative picture of a recurrent GCT in the thoracic spine, approached anteriorly

We planned our surgical approach based on a variety of factors including tumor location, extent, area of cord compression, degree of instability and status of previous implants. In all, of the nine surgical interventions, four were done by the anterior approach [Figure 1]. These had ‘pure’ anterior lesions (anterior and middle column affection), with the posterior column structurally strong either due to the absence of disease or due to previous surgical reconstruction. Two surgeries were done by the posterior approach. One of these had posterior element affection, while one had a recurrent posterior disease (probably residual tumor after the primary surgery) with an infected wound. Three had a combined posterior and anterior surgery [Figure 2]. These were the cases with three-column affection (two cases) or posterior and middle column affection (one case).

**Figure 2A:**
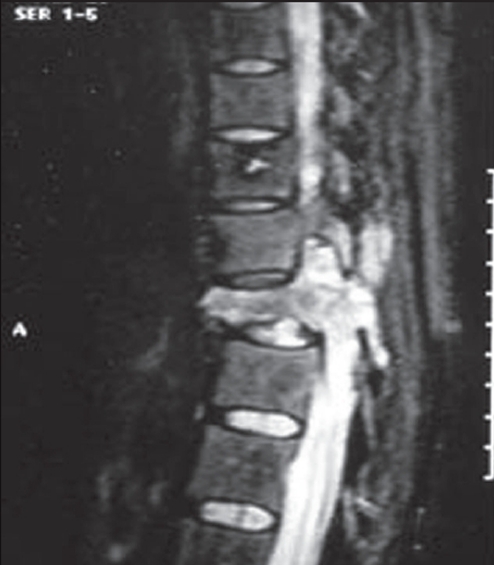
MRI appearance of a GCT in the thoracic spine. Note the lesion extending through the pedicle, affecting all three columns

**Figure 2B:**
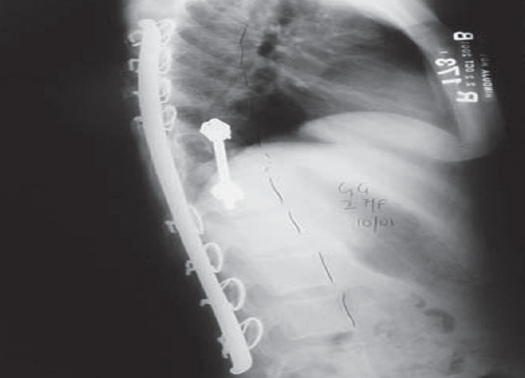
Postoperative X-ray in the same patient after combined posterior and anterior resection and reconstruction

For all cases done posteriorly, we used the trans pedicular route for tumor resection and reconstruction with posterior fixation by pedicle screws or sub laminar wires. We found decompression to be adequate by this approach and it also gave enough access for reconstruction. Our earlier four cases had reconstruction with tricortical iliac strut grafts, while we used cages and bone cement in the three later cases. Two cases, done by the ‘posterior only’ approach (details as above) did not need reconstruction.

All our nine interventions were preceded by digital subtraction angiography (DSA) embolization to reduce intraoperative bleeding. None of our surgeries had to be cut short due to uncontrolled bleeding.

Four of our patients, all with recurrent GCTs received radiotherapy postoperatively. The doses were varied depending on the type of lesion and previous radiotherapy.

## RESULTS

Our eight interventions achieved the short-term goals of neurological spinal decompression and stabilization. All eight returned to Frankel Grade E, in the early postoperative period. The ninth intervention patient had worse neurology (Frankel C) to begin with, took longer (three months on an average) to come back to Frankel Grade E.

We had no major complications, though two of the recurrent cases had superficial wound problems that healed uneventfully. In the long follow-up, two of our cases had recurrence of the GCT. One 23-year-old female (case number 1) with a back and front surgery done in 1997 returned with a recurrence in 2002, with signs of cord compression and needed anterior surgical decompression and reconstruction with a cement spacer. The other, a 36-year-old male (case number 5), was operated for a GCT of the C5 vertebral body, by anterior corpectomy and bone grafting by the senior author (SYB) in 1993. He did not receive postoperative radiation therapy and the tumor recurred after a year. This time, he had a complex back and front reconstruction with backup radiotherapy, which ensured a long tumor-free period. The ‘aggressive’ GCT, however, recurred six years postoperatively and a redo anterior reconstruction with fibular graft and plating was performed in 1999. Further radiation could not be given postoperatively due to potential danger of radiation myelopathy, as maximum possible doses had been previously received.

Both these patients have remained asymptomatic till the time of submission of this paper (2006), one for five years and the other for seven years. Both have been followed up regularly and with a close watch on recurrences.

## DISCUSSION

Giant cell tumor in the spine, especially above the sacrum, is a relatively rare entity.^3^ According to the report from the Istituto Ortopedico Rizzoli, the incidence is 2.9% in the vertebrae above the sacrum and 2.5% in the sacrum in all giant cell tumors of the bone.^4^

We report nine surgical interventions in six patients of the GCT of the spine, from the 200 spinal tumors operated by us in the 13 years between 1993 and 2006. Though the incidence is similar to published data, there were no sacral GCTs in our series.

Spinal GCTs most commonly present with pain due to the expansile lesion with or without vertebral collapse and spinal instability.^5^ This often gets compounded by neurological deficit due to encroachment onto the spinal canal.^6^ Asymptomatic, incidental radiological occurrence is uncommon in spinal GCTs. All our patients presented with spinal instability and varying grades of cord compression.

Radiologically, GCTs of the spine present as cystic, expansile lesions on plain roentgenograms.^7^ ‘Soap bubble’ appearance has been described. As against an aneurysmal bone cyst (ABC), a GCT usually affects the vertebral body. Soft tissue outside the cyst is often seen on CT/ MR scans and seems to suggest local aggression. This soft tissue could be misinterpreted as infection and is usually not seen in ABCs. Differential diagnosis of spinal GCTs, on clinico-radiological evidence remains ABC and tuberculosis (TB). Definitive diagnostic biopsy before treatment should be performed.

For the previously cited reasons, none of our cases had a positive presurgical histology via biopsy (though the recurrences had histopathogical diagnosis from the material from the first surgery). However, a transpedicular, CT-guided core needle biopsy is definitely recommended in all cases and is the best method of obtaining a pretreatment diagnosis. We have been able to get histological diagnosis in 80-85% cases in our clinical practice. Procedure site bleeding is not uncommon and the interventional radiologist should be aware of this.^8^ If biopsy is nonconclusive, an intraoperative frozen section becomes mandatory.

Pathologists can usually comment on the aggressiveness of the GCT, which helps planning treatment. This pertains especially to the postoperative use of radiotherapy and the frequency of postoperative CT scans. In tumors reported as ‘aggressive’ we plan radiotherapy post resection even if the resection has been adequate. Also, in these cases, CT scans are performed every six months for two years at least, to keep a watch on possible recurrences.

Giant cell tumor being a highly vascular tumor, DSA aided tumor embolization within 24 hours before surgery is recommended.^9^ This not only minimizes blood loss, but also permits the surgeon a dry field to carry out optimum tumor excision. In some cases however, DSA shows that a common vascular feeder supplies the spinal cord as well as the tumor. In such cases, embolization cannot be carried out due to the risk of vascular infarct to the spinal cord.^1^

Complete, extralesional surgical resection would be the ideal treatment for spinal GCTs.^10^ However, as the tumor is extremely close to important neurovascular structures and has usually broken through the cortex by the time it is diagnosed, extralesional / en bloc resection is generally not possible. Thus marginal or intralesional resection followed by local radiotherapy is the treatment that is usually resorted to. Thorough intralesional curettage and meticulous excision of as much tumor as possible, is important. The tumor wall, rather than the tumor ‘substance’ has the diagnostic features on histology / frozen section.

Usually, some microscopic tumor tissue is expected to stay behind, however thorough the surgical excision is and hence postoperative radiotherapy is recommended. Though earlier literature seemed to suggest that irradiation converts benign GCTs to malignant ones, this is no longer true with modern radiotherapy techniques, especially by keeping the total radiation dose under 50Gy.^2^ ^11^ ^12^

Recommended method of reconstruction is by cement or metallic cages. Bone graft is avoided because the tumor is known to recur in the grafted bone. Also postoperative irradiation is frequently used, hampering graft fusion.^2^ ^11^ ^12^ Local recurrence in the spine is reported to be lower compared with other locations. A study of the natural history of GCTs of the spine showed that patients with spinal lesions have a better prognosis than nonspine GCTs.^13^ Fewer local recurrences, metastasis and redo surgeries have been reported in spinal GCTs as against GCTs in other areas. Boriani *et al.* 5 10, in their study of incidence of tumor recurrence in spinal GCTs, state that recurrence rates were substantially higher among patients treated with attempted surgical excision before referral to a tertiary care center. They also report that recurrences were higher in GCTs that involved the vertebral body and posterior elements compared to those limited to the vertebral body only. Higher recurrences were reported in tumors that had extra-osseous extension into the canal and into the paraspinous musculature.^3^

A high index of suspicion is mandatory to diagnose recurrences in the GCTs.^7^ ^9^ Symptomatology, clinical examination as well as imaging modalities should be made use of in the best possible manner and with an optimum frequency. If suspected, a biopsy may be done to prove this, unless it is very obvious on the CT / MRI. Herein lies the importance of baseline postoperative radiological imaging to compare against later scans. In our group, five of the nine surgeries were for tumor recurrence. Keeping that in mind, we routinely perform a one-yearly CT scan to monitor the tumor status and recommend postoperative irradiation to minimize recurrence rates.

To summarize, spinal GCT are challenging clinical entities. Surgical intervention is mandatory and demanding and close follow-up is important to spot recurrences early.
